# Increased risk of infection with SARS-CoV-2 Omicron BA.1 compared with Delta in vaccinated and previously infected individuals, the Netherlands, 22 November 2021 to 19 January 2022

**DOI:** 10.2807/1560-7917.ES.2022.27.4.2101196

**Published:** 2022-01-27

**Authors:** Dirk Eggink, Stijn P Andeweg, Harry Vennema, Noortje van Maarseveen, Klaas Vermaas, Boris Vlaemynck, Raf Schepers, Arianne B van Gageldonk-Lafeber, Susan van den Hof, Chantal BEM Reusken, Mirjam J Knol

**Affiliations:** 1 Center for Infectious Disease Control, WHO COVID-19 reference laboratory, National Institute for Public Health and the Environment (RIVM), Bilthoven, The Netherlands; 2 Saltro Diagnostic Center for Primary Care, Utrecht, The Netherlands; 3 Department of Medical Microbiology, University Medical Center Utrecht, Utrecht, the Netherlands; 4 SYNLAB, Heppignies, Belgium

**Keywords:** air-borne infections, viral infections, severe acute respiratory syndrome - SARS, vaccines and immunisation, epidemiology, laboratory, SARS-CoV-2 Omicron Variant of Concern Vaccine escape

## Abstract

Infections with the Omicron SARS-CoV-2 variant are rapidly increasing worldwide. Among 174,349 SARS-CoV-2-infected individuals (≥ 12 years), we observed an increased risk of S gene target failure, predictive of the Omicron variant, in vaccinated (odds ratio (OR): 3.6; 95% confidence interval (CI): 3.4–3.7) and previously infected individuals (OR: 4.2; 95% CI: 3.8–4.7) compared with infected naïve individuals. This suggests vaccine- or infection-induced immunity against SARS-CoV-2 infections is less effective against the Omicron than the Delta variant.

On 26 November 2021, the World Health Organization (WHO) declared the Omicron variant (Phylogenetic Assignment of Named Global Outbreak Lineages (Pangolin) designation B.1.1.529) of severe acute respiratory syndrome coronavirus 2 (SARS-CoV-2) a variant of concern [1]. Globally, infections caused by Omicron have increased rapidly, showing higher transmissibility of this variant [2,3]. Omicron became the dominant variant in parts of Europe in early 2022 [4]. It displays a number of alterations in the spike protein, including around 30 amino acid substitutions, three deletions and one insertion [5-7]. These alterations raise concerns about the protection evoked by current SARS-CoV-2 vaccines against the Omicron variant.

To explore possible escape from vaccine- or infection-induced immunity by Omicron compared with Delta, we here investigate the distribution of the Delta (B.1.617.2) and Omicron variants among SARS-CoV-2-positive individuals who had been naïve, had received the complete primary vaccination series or had a previous infection. We employed a case-only approach in which we compared the immune status among cases infected with the Omicron vs the Delta variant. We thereby assess the relative effectiveness of vaccination against Omicron vs Delta viruses [8]. Similarly, we analyse if the protective effect of previous SARS-CoV-2 infection is different against a new infection with Delta vs Omicron viruses. 

## S gene target failure as a measure for Omicron infection

We used data from two large diagnostic laboratories analysing specimens from national community testing in the Netherlands that make use of the TaqPath COVID-19 RT-PCR kit (ThermoFisher Scientific, Nieuwegein, The Netherlands). The Omicron (BA.1) but not the Delta variant, with the exception of some very sporadically circulating Delta variant strains, possesses a deletion at amino acid positions 69 and 70 of the spike protein (Δ69–70) that has been associated with failure of the probe targeting the S gene, while the ORF1ab and N probes result in a positive signal (S gene target failure (SGTF), also referred to as S drop-out). This failure of detection of the S gene target in an otherwise positive PCR test has proven to be a highly specific proxy for the presence of the Alpha variant (B.1.1.7) in the past and also identifies Omicron [9-12]. However, with lower viral loads, the S gene target tends to be the least sensitive of the three targets in the Taqpath system, which could result in incorrect interpretation of an SGTF result. To avoid false-positive SGTF results, we used a stringent threshold to identify likely Omicron infections. In-house titration showed decreased sensitivity for the S gene target in case of viral loads that gave a quantification cycle (Cq) value of around 32 or more for the ORF1ab and N targets. Therefore, only results with a Cq value ≤ 30 for both targets were included for further analyses.

Sequencing of a random selection of SGTF samples included in this study confirmed the presence of the Omicron variant (BA.1) in 325 of 329 cases (98.8%). Alpha (n = 1) and Delta (n = 3) variants containing the Δ69–70 were detected in the remaining samples. The correlation between SGTF and Omicron increased over time, with only Omicron detections in the SGTF samples after 5 December 2021.

S gene testing results were linked to vaccination status data from the national notification register (OSIRIS), which includes all SARS-CoV-2 cases in the Netherlands. If vaccination status was unknown from the notification register, we used self-reported vaccination status from the community testing register (CoronIT). A person was defined as having completed the primary vaccination series when they had received two doses of Comirnaty (BNT162b2, BioNTech/Pfizer, Mainz, Germany/New York, United States (US)), Spikevax (mRNA-1273, Moderna, Cambridge, US) or Vaxzevria (ChAdOx1, AstraZeneca, Cambridge, United Kingdom) more than 14 days before the symptom onset date or one dose of COVID-19 Vaccine Janssen (Janssen-Cilag International NV, Beerse, Belgium) more than 28 days before the symptom onset date. For asymptomatic cases, we used sample date (6,165 of 110,965 vaccinated cases) and for symptomatic cases or unknown symptoms status for whom onset date was missing, we used the sample date minus 2 days (19,136 of 110,965 vaccinated cases). People who had not received any vaccine were defined as unvaccinated. Previous infection was defined as a positive PCR or antigen test result at least 8 weeks before the current positive test, based on the national notification and testing register. We excluded children younger than 12 years because they had not been eligible for vaccination in the study period and there had been different testing policies in this age group over time, influencing the likelihood of detecting a previous infection.

## Statistical analysis

Among infected individuals, we compared the percentage of SGTF results between unvaccinated cases without a known previous infection (naïve), primary vaccinated cases without a known previous infection, and unvaccinated cases with a known previous infection. The number of primary vaccinated persons with a previous infection was small and therefore excluded. We performed logistic regression to estimate the association between immune status and SGTF, adjusting for testing date, 10-year age group and sex. In addition, we stratified the analyses by three age groups (12–29, 30–59 and ≥ 60 years) and adjusted for testing date, 5-year age group and sex. If the Omicron and Delta variants had similar ability to escape immunity from vaccination or previous infection, we would expect the same proportion of SGTF in vaccinated or previously infected persons as in naïve individuals, i.e. an odds ratio (OR) of 1. As travel history could be related to being infected with the Omicron variant and with being vaccinated, we performed an additional analysis where we excluded cases with a history of travel outside the Netherlands in the 14 days before symptom onset.

## Immune status and S gene target failure

Between 22 November 2021 and 19 January 2022, 174,349 PCR-positive samples with a Cq value ≤ 30 for ORF1ab and N gene targets were analysed for SGTF and included in the current analysis. SGTF was present in 80,615 cases (46.2%); the percentage of SGTF increased rapidly from early December (Figure). 

**Figure:**
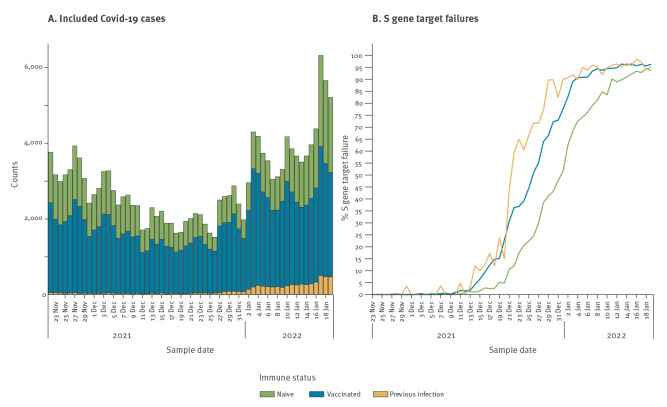
Number of included COVID-19 cases and percentage of S gene target failures by immune status, the Netherlands, 22 November 2021–19 January 2022 (n = 174,349)

COVID-19: coronavirus disease.

Cases with SGTF were younger and more often had a travel history (Table 1). Of the SGTF cases, 53,291 (66.1%) had received primary vaccination compared with 61.5% (n = 57,674) of non-SGTF cases, and 6.5% (n = 5,253) had a previous infection compared with 1.4% (1,295) of the non-SGTF cases. Correspondingly, we found an adjusted OR (AOR) of 3.6 (95% confidence interval (CI): 3.4–3.7) for the association between full vaccination and SGTF (Table 2). The AOR for previous infection was 4.2 (95% CI: 3.8–4.7). When excluding cases with any known travel history, the OR remained the same for previous infection (OR = 4.2; 95% CI: 3.8–4.7) and was slightly lower but still significant for vaccination (OR = 3.4; 95% CI: 3.2–3.5).

**Table 1:** Characteristics of SARS-CoV-2 cases 12 years and older with S gene detected and S gene target failure, the Netherlands, 22 November 2021–19 January 2022 (n = 174,349)

Total	S gene detected		S gene target failure	
	n	%	n	%
	93,734	100	80,615	100
Immune status				
Naïve	34,765	37.1	22,071	27.4
Primary vaccination	57,674	61.5	53,291	66.1
Previous infection	1,295	1.4	5,253	6.5
Age group (years)				
12–19	11,338	12.1	21,231	26.3
20–29	13,907	14.8	22,676	28.1
30–39	18,028	19.2	13,554	16.8
40–49	16,871	18.0	9,815	12.2
50–59	13,872	14.8	9,223	11.4
60–69	11,427	12.2	3,009	3.7
70–79	6,501	6.9	941	1.2
≥ 80	1,790	1.9	166	0.2
Sex				
Male	47,172	50.3	39,981	49.6
Female	46,562	49.7	40,634	50.4
Travel history				
Yes	1,000	1.1	4,162	5.2
No	43,630	46.5	21,363	26.5
Unknown	49,104	52.4	55,090	68.3
Interval	Median	IQR	Median	IQR
Number of days between onset and last vaccination	155	130–183	157	139–180
Number of days between onset and previous infection	343	246–396	274	170–390

IQR: interquartile range; SARS-CoV-2: severe acute respiratory syndrome coronavirus 2.

**Table 2:** Association between immune status and S gene target failure, adjusted for day of sampling, sex and 10-year age group, the Netherlands, 22 November 2021–19 January 2022 (n = 174,349)

Immune status	All data	Without travel history
	OR (95% CI)	OR (95% CI)
**Naïve**	Reference	Reference
**Primary vaccination**	3.6 (3.4–3.7)	3.4 (3.2–3.5)
**Previous infection**	4.2 (3.8–4.7)	4.2 (3.8–4.7)

CI: confidence interval; OR: odds ratio.

Stratified by age group, the AOR for primary vaccination were 4.1 (95% CI: 3.9–4.4), 3.2 (95% CI: 3.0–3.4) and 2.8 (95% CI: 2.3-3.2) for 12–29 years, 30–59 years and ≥ 60 years, respectively. For previous infection, the AOR were 3.7 (95% CI: 3.2–4.3), 4.8 (95% CI: 4.0–5.7) and 6.6 (95% CI: 3.5–13.0) for 12–29 years, 30–59 years and ≥ 60 years, respectively.

## Ethical statement

The Centre for Clinical Expertise at the National Institute for Public Health and the Environment (RIVM) assessed the research proposal following the specific conditions as stated in the law for medical research involving human subjects. The work described was exempted for further approval by the ethical research committee. Pathogen surveillance is a legal task of the RIVM and is carried out under the responsibility of the Dutch Minister of Health, Welfare and Sports. The Public Health Act (Wet Publieke Gezondheid) provides that RIVM may receive pseudonymised data for this task without informed consent.

## Discussion

Our results suggest a large reduction in protection against SARS-CoV-2 infection with the Omicron variant, as measured by SGTF, compared with the Delta variant after primary vaccination. This is in accordance with in vitro studies that showed a 30–40-fold reduction in neutralisation of the Omicron variant compared with wild type when using convalescent sera or sera of individuals who had completed the primary vaccination series [5-7]. Several studies have reported a substantial reduction in vaccine effectiveness against infection with the Omicron variant compared with Delta after primary vaccination [13-16]. After booster vaccination, the vaccine effectiveness against infection with the Omicron variant increased but was still lower than against Delta [14-18]. Studies have shown that vaccine effectiveness against severe COVID-19 with the Omicron variant is very high after booster vaccination, although lower than against severe COVID-19 with Delta [19,20]. 

We showed that individuals with a previous infection had a higher risk of infection with Omicron than with Delta compared with naïve individuals, suggesting that previous infection with another SARS-CoV-2 variant provides lower levels of protection against Omicron than against Delta infection. This contrasts with what we observed in a previous analysis, where we did not find an increased risk of infection with the Delta, Beta (B.1.351) and Gamma (P.1) variants vs the Alpha variant in previously infected individuals [21]. Our finding of reduced protection against reinfection with Omicron is in line with reports showing increased risk of reinfections with the Omicron variant [22-24].

We found a lower OR for vaccination in older age groups, suggesting that the difference in vaccine effectiveness against Omicron versus Delta is lower with higher age. We do not have an explanation for this observation and therefore this may need further investigation. 

Our study looked at infections based on community testing. Several studies suggest that infection with the Omicron variant causes less severe disease, with lower rates of hospitalisations and intensive care admission than for Delta infection, also when considering the effect of vaccination and previous infection [12,25,26]. The effectiveness of primary vaccination against hospitalisation with Omicron infection was reported to be around 50% at 25 weeks or more after vaccination, and the effectiveness increased to ca 90% within 2–9 weeks after booster vaccination [19,20]. Even with lower disease severity for Omicron compared with Delta, the higher transmissibility will probably lead to more hospital admissions if no additional measures are taken.

A strength of a case-only design is that it prevents bias from poor control selection and it prevents bias from exposure misclassification differential by disease status, as it only includes people with the disease. The method we used is recommended by the WHO to assess the impact of new variants on vaccine effectiveness [8]. Our study also has some limitations. We relied on reported previous infections, and therefore some misclassification will have occurred as not all previous infections are detected and hence reported. Also using self-reported vaccination status may have led to some misclassification, although it is unlikely that this would be different in people infected with the Omicron compared with the Delta variant. We used SGTF as a proxy for Omicron. Omicron was confirmed by sequencing in 99% of 329 samples, so limited misclassification due to use of SGTF can be expected to be limited. Of note, SGTF can discriminate between Delta and the BA.1 but not the BA.2 variant of Omicron. At the end of our study period, BA.2 had only been rarely detected in the Netherlands. For our study design to be valid, we have to assume that vaccination and previous infection are independent of variant status, i.e. there should not be a third factor influencing both vaccination and having an Omicron infection. This is questionable for people with a travel history, as vaccination might be a requirement to allow travel. A sensitivity analysis excluding cases with any travel history did not substantially change our results. We have not yet been able to assess the effect of booster vaccination which has only recently started in the Netherlands (18 November 2021). 

## Conclusion

Our results suggest a large decrease in protection from vaccine- or infection-induced immunity against SARS-CoV-2 infections caused by the Omicron variant compared with the Delta variant. This emphasises the need of booster vaccination and will warrant implementing non-pharmaceutical interventions to prevent overwhelming hospital care if COVID-19 severity is not reduced to a great extent.
